# Interactions of Salmonella enterica Serovar Typhimurium and Pectobacterium carotovorum within a Tomato Soft Rot

**DOI:** 10.1128/AEM.01913-17

**Published:** 2018-02-14

**Authors:** Andrée S. George, Clayton E. Cox, Prerak Desai, Steffen Porwollik, Weiping Chu, Marcos H. de Moraes, Michael McClelland, Maria T. Brandl, Max Teplitski

**Affiliations:** a Soil and Water Science Department, Genetics Institute, University of Florida-IFAS, Gainesville, Florida, USA; b Department of Microbiology and Molecular Genetics, University of California, Irvine, Irvine, California, USA; c Produce Safety and Microbiology Research Unit, Western Regional Research Center, Agricultural Research Service, U.S. Department of Agriculture, Albany, California, USA; University of Manchester

**Keywords:** food safety, *Pectobacterium*, produce, *Salmonella*, microbe-microbe interactions, transposons

## Abstract

Salmonella spp. are remarkably adaptable pathogens, and this adaptability allows these bacteria to thrive in a variety of environments and hosts. The mechanisms with which these pathogens establish within a niche amid the native microbiota remain poorly understood. Here, we aimed to uncover the mechanisms that enable Salmonella enterica serovar Typhimurium strain ATCC 14028 to benefit from the degradation of plant tissue by a soft rot plant pathogen, Pectobacterium carotovorum. The hypothesis that in the soft rot, the liberation of starch (not utilized by *P. carotovorum*) makes this polymer available to Salmonella spp., thus allowing it to colonize soft rots, was tested first and proven null. To identify the functions involved in Salmonella soft rot colonization, we carried out transposon insertion sequencing coupled with the phenotypic characterization of the mutants. The data indicate that Salmonella spp. experience a metabolic shift in response to the changes in the environment brought on by Pectobacterium spp. and likely coordinated by the *csrBC* small regulatory RNA. While *csrBC* and *flhD* appear to be of importance in the soft rot, the global two-component system encoded by *barA sirA* (which controls *csrBC* and *flhDC* under laboratory conditions) does not appear to be necessary for the observed phenotype. Motility and the synthesis of nucleotides and amino acids play critical roles in the growth of Salmonella spp. in the soft rot.

**IMPORTANCE** Outbreaks of produce-associated illness continue to be a food safety concern. Earlier studies demonstrated that the presence of phytopathogens on produce was a significant risk factor associated with increased Salmonella carriage on fruits and vegetables. Here, we genetically characterize some of the requirements for interactions between Salmonella and phytobacteria that allow Salmonella spp. to establish a niche within an alternate host (tomato). Pathways necessary for nucleotide synthesis, amino acid synthesis, and motility are identified as contributors to the persistence of Salmonella spp. in soft rots.

## INTRODUCTION

Although traditionally associated with products of animal origin, over the last decade, multistate outbreaks of nontyphoidal Salmonella spp. associated with produce highlight the need to further understand the ecology of this human pathogen in alternate hosts, such as plants. Despite advances in understanding how this pathogen contaminates produce (1, 2), salmonellosis outbreaks linked to the consumption of fresh fruits and vegetables continue to present a global problem. Interactions between Salmonella spp. and the native microbial communities are hypothesized to contribute to the ability of this human pathogen to colonize plants (3–8). It has been reported that Salmonella enterica strains may benefit from the presence of plant pathogens, such as Pectobacterium carotovorum, *Dickeya dadantii*, and Xanthomonas spp. (3, 7, 9). These bacteria cause disease on various plants, including leafy greens and tomatoes, and represent a risk factor for the increased likelihood of produce contamination with enterics (4, 6, 7). Several mechanisms underlying these interactions have been examined. Quorum sensing signal exchange between Salmonella and Pectobacterium spp., changes in the response of Salmonella spp. to the environment and the uptake of nutrients from degraded plant tissue have all been tested, although none of them fully account for the ability of Salmonella spp. to efficiently colonize lesions created by phytopathogens (3, 10–12).

Interactions with other bacteria are an important factor in the successful colonization of plant surfaces and tissues by Salmonella spp., although the importance to internal colonization remains understudied (1, 3). One of the tested hypotheses was that Salmonella spp. sense production of the population density-dependent signals by other bacteria within the soft rot and respond accordingly (10). While Salmonella spp. are known to possess the ability to detect *N*-acyl homoserine lactone (AHL) bacterial quorum sensing signals via the LuxR homologue SdiA (10, 13, 14), the exact function of this protein remains elusive (15, 16). While Salmonella spp. detected AHLs from Pectobacterium strains *in vitro*, the *sdiA* gene was expressed at a low level inside the tomato fruit or the soft rot, and, consequentially, the deletion of this regulator did not significantly impact fitness within soft rots (10). The deletion of the Salmonella second quorum sensing (QS) system mediated by the autoinducer 2 (AI-2) had no effect on growth in tomatoes with or without Pectobacterium carotovorum, providing further evidence that these two QS-mediated signal exchanges are not a driving force of this interaction (11).

Nutrient exchange or environmental change due to soft rot likely explains the growth benefit gained by Salmonella species. Transcriptomic studies of Salmonella spp. in *D. dadantii* soft rot lesions on cilantro and lettuce have shown that Salmonella spp. use distinct metabolic pathways and take advantage of the substrates and physicochemical conditions that result from the maceration of leaf tissue by soft rotters (7). While Salmonella spp. clearly benefit from the presence of Pectobacterium spp., the mechanisms behind this phenomenon remain unclear. The process by which the plant cell wall-degrading enzymes (PCWDEs) of Pectobacterium spp. macerate plant tissue is well established (17–19). Changes in macerated plant tissue, such as a decrease in pH (20), the freeing of carbohydrate monomers and of starch (which is not metabolized by Pectobacterium spp.), and regulation of plant defenses in the early stages of infection create an environment in which Salmonella spp. are able to thrive (6, 21). Indeed, it has been shown that damage to plants, whether mechanical or microbial, enhances colonization by enteric pathogens (22). The release of nutrients from plant tissues provides a means for the survival for soft rot plant pathogens, such as Pectobacterium carotovorum (18, 19, 23). Salmonella spp., which do not possess PCWDEs, may scavenge the products released by the enzymatic activity of Pectobacterium species. Previously, we established that the deletion of the *kdgR* gene, which codes for a repressor of cell wall degradation and uptake of monomers and dimers resulting from the plant cell wall breakdown, was beneficial to the growth of S. enterica serovar Typhimurium strain ATCC 14028 (*S*. Typhimurium 14028) in soft rot (24). However, further investigation of the KdgR regulon did not offer a conclusive explanation for its role in the ability of Salmonella spp. to benefit from the presence of Pectobacterium species.

To obtain a more comprehensive picture of the biology of Salmonella spp. in Pectobacterium soft rot, we made use of a transposon-derived mutant library which we screened for both deleterious and beneficial mutations. This transposon insertion analysis indicates that motility and amino acid and nucleotide synthesis are important for the growth of *S*. Typhimurium 14028 in soft rot.

## RESULTS

### Plant maceration, and not the presence of Pectobacterium carotovorum *per se*, provides a benefit to *S*. Typhimurium 14028.

**FIG 1:**
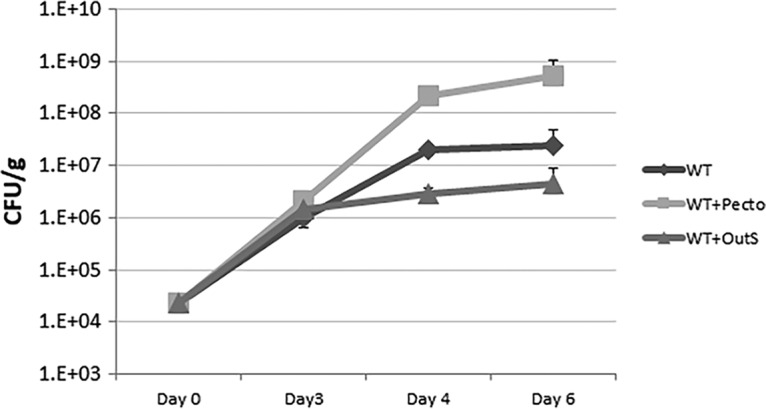
Growth curves of wild-type Salmonella in tomatoes. Tomatoes were treated with no Pectobacterium carotovorum. (wild type [WT]), with Pectobacterium carotovorum (WT + Pecto), and with a Pectobacterium Δ*outS* mutant (WT + OutS). Cells were plated on xylose lysine deoxycholate (XLD) agar in triplicate (*n* = 6 to 9) in and counted after 3, 4, and 6 days. Error bars reflect the standard error. Final concentrations are significantly different (*P* < 0.05).

As shown in previous reports, Salmonella enterica serovar Typhimurium strains reach higher cell numbers in tomatoes macerated by the plant pathogens of Pectobacterium than in intact tomatoes (6, 10, 12). To test the hypothesis that *P. carotovorum* degradation of plant tissue, and not the presence of the plant pathogen *per se*, promoted the growth of *S*. Typhimurium 14028, we constructed an *outS* mutant in Pectobacterium carotovorum strain WP114. This mutation results in bacteria that are unable to secrete the enzymes responsible for the breakdown of the plant cell wall (25). Expectedly, the resulting *P. carotovorum* mutant is unable to effectively secrete pectate lyases. In tomatoes infected with the *P. carotovorum outS* mutant, there were no visible signs of the characteristic soft rot disease progression (see Fig. S1 in the supplemental material). This observation is consistent with the previous report of the significantly reduced virulence of *P. carotovorum outS* mutants in other strains of this pathogen (26). Prior to the appearance of disease symptoms caused by the wild-type Pectobacterium bacterium (day 3), *S*. Typhimurium 14028 reached ∼10^6^ CFU/g in all three sample groups: tomatoes only, tomatoes infected with *P. carotovorum* SR38, and tomatoes with the *P. carotovorum outS* mutant (Fig. 1). However, once soft rot symptoms developed in tomatoes infected with wild-type *P. carotovorum* SR38, the populations of *S*. Typhimurium 14028 reached 10^9^ CFU/g. In contrast, the growth of *S*. Typhimurium 14028 in tomatoes that were free of Pectobacterium carotovorum reached a maximum density of about 10^7^ CFU/g; in tomatoes coinfected with *S*. Typhimurium 14028 and an *outS* mutant of *P. carotovorum*, the population sizes of *S *. Typhimurium 14028 did not occur to the same extent and were ∼100-fold less than those in soft rotted tomatoes and 10-fold less than those in the Salmonella-only control tomato (Fig. 1). Statistical analysis showed that final population sizes were significantly different from each other (*P* < 0.05). This test supported the null hypothesis that plant maceration, and not the presence of Pectobacterium carotovorum *per se*, leads to substantial growth increase of *S*. Typhimurium 14028.

### Salmonella starch utilization genes are upregulated but not required for fitness within soft rots.

**FIG 2:**
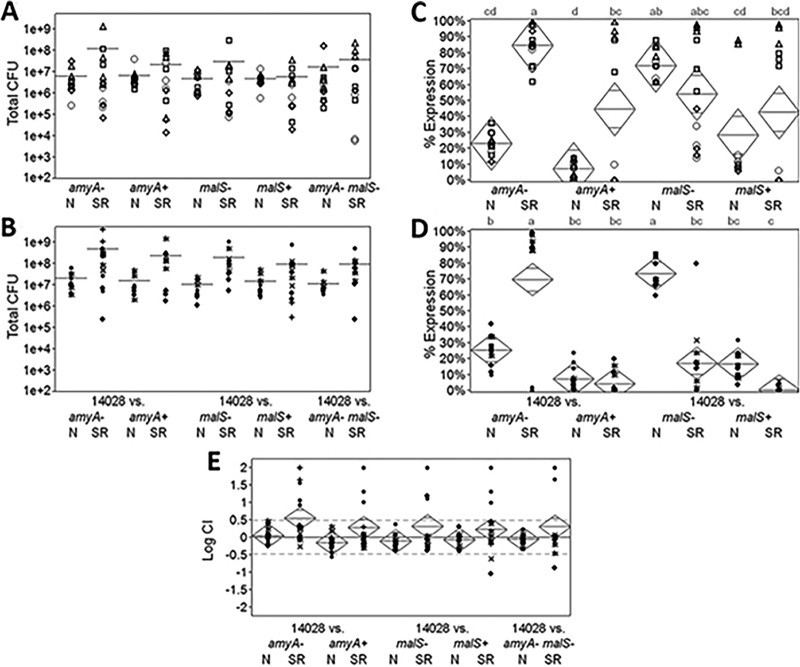
Function of *amyA* and *malS. In vivo* assays in normal (N) or soft-rotted (SR) green tomatoes. (A) Single infections of marked α-amylase mutants. *n* ≥ 11. (B) Coinfections between marked α-amylase mutants and WT *S*. Typhimurium 14028. *n* ≥ 9. Bars show group means. Different markers represent samples from the same independent experiments. No differences were significant, as determined by the Tukey honestly significant difference (HSD) test. (C) Percent resolution of *tetR* in RIVET reporter promoter-*tnpR* (− mutants) or gene-*tnpR* (+ mutants) fusions in single infections with α-amylase mutants only. *n* = 12. (D) Percent resolution of *tetR* in RIVET reporters in coinfections with marked α-amylase mutants and WT *S*. Typhimurium 14028. *n* ≥ 12. (E) Competitive fitness between WT *S*. Typhimurium 14028 and marked α-amylase mutants during coinfections. Diamonds show means and 95% confidence interval bars at the top and bottom. Different markers represent samples from the same independent experiments. Lowercase letters identify significance groups, as determined by the Tukey HSD test.

*P. carotovorum* is known to liberate amylose and amylopectin as a consequence of the degradation of the integrity of plant cells. *P. carotovorum* lacks the ability to utilize starch (27, 28). Because Salmonella spp. are capable of digesting starch (29), which can be up to 10% dry weight in mature green tomatoes (30, 31), we tested the hypothesis that this human pathogen benefits from the soft rot due to its ability to utilize starch that is liberated and unused by *P. carotovorum*. This approach was two-pronged and involved first an assessment of the expression of the *Salmonella malS* and *amyA* genes involved in starch utilization, and second, a determination of the fitness of corresponding mutants in intact and macerated tomato fruit tissue. In order to assess the expression, recombination-based *in vivo* expression technology (RIVET) was employed (32). Expression is measured as the percentage of cells which become susceptible to tetracycline as a result of the activation of the promoter of interest and the associated excision of the tetracycline cassette-containing reporter marker. As shown in Fig. 2, RIVET revealed that *amyA* was expressed only modestly in intact tomatoes (5 to 20%) but strongly (70 to 100%) in soft rots. The strongest induction was observed in *amyA* mutants, suggesting the presence of a feedback mechanism that likely depends on the availability of either the substrate or the degradation products. The basal expression level of *malS*, which encodes a periplasmic α-amylase (33), was higher than that of *amyA*. In intact tomatoes, *malS* was expressed at 5 to 80%, while expression in soft rotted tomatoes was generally lower, although more varied. As with *amyA*, the expression patterns of *malS* suggest feedback regulation, as the expression of the *malS* RIVET reporter was highest in the *malS* mutant background. We observed some variability in the activation of *amyA* and *malS* reporters in soft rots, perhaps representative of the inherent heterogeneity of this environment. We then tested the fitness of the Salmonella *amyA* and *malS* mutants in soft rots and in intact green tomatoes compared to that of the wild-type Salmonella bacteria. As shown in Fig. 2E, there were no significant differences in the fitness of the mutants under any of the tested conditions. Therefore, the hypothesis that the benefits derived by Salmonella spp. from soft rot disease are due to their ability to scavenge starch via *amyA* and *malS* was proven null.

### Transposon insertion analysis reveals differences between metabolic pathways necessary for growth in the intact and soft rot tomatoes.

Having previously reported that *kdgR* alone is not responsible for the growth of *S*. Typhimurium 14028 in soft-rotted tomatoes (24) and in light of the fact that the starch-scavenging hypothesis was proven null, we performed a high-throughput assay in order to identify the full complement of genes responsible for this interaction. Transposon sequencing (Tn-Seq) has become an important tool for untangling complex metabolic networks in Salmonella spp. in a variety of habitats (34, 35). Here, we use a similar technique, transposon insertion analysis, to identify mutations that affect the ability of *S*. Typhimurium 14028 to grow in green tomatoes with and without soft rot.

In plants, enteric pathogens rely on a distinct set of genes in order to be competitive against the native microbiota (7, 36, 37). Libraries of *S*. Typhimurium 14028 mutants constructed with tagged transposons were seeded into intact green tomatoes, green tomatoes rotted with Pectobacterium carotovorum, and green tomatoes inoculated with a *Pectobacterium outS* mutant. Generally, similar Salmonella functions appear to be involved in the persistence within the intact tomatoes and those infected with the *outS* mutant (Fig. S2). This further supports the assertion that the soft rot, and not the presence of Pectobacterium cartovorum, is responsible for the observed growth increase. Overall, there was a large number of mutations that reduced Salmonella fitness in the soft rot and nearly as many mutants that benefited from the soft rot. Indeed, ∼600 mutants (∼54%) were less competitive in the soft rot than in the intact tomato, and ∼500 mutants (∼46%) (false-discovery rate [FDR], <0.05) were more competitive than the wild-type strain (Fig. S3).

**FIG 3:**
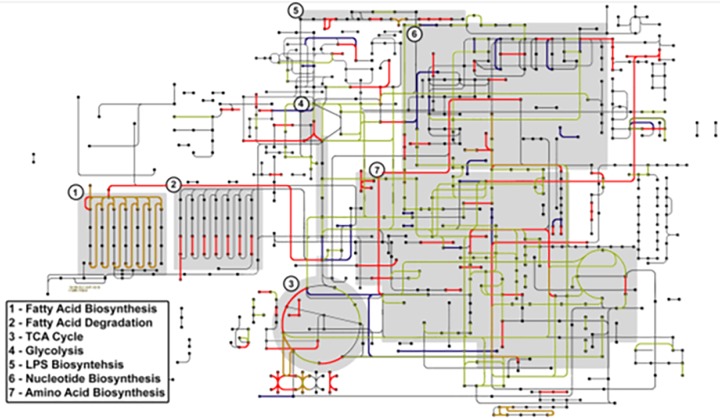
Salmonella metabolic pathways affected by soft rot. Map of Salmonella metabolism downloaded from the Kyoto Encyclopedia of Genes and Genomes (KEGG) constructed from transposon insertion analysis (TIA) data. Lines represent genes under negative selection in the soft rot (blue), intact fruit, (red) and both conditions (green).

The transposon mutant screen revealed that most insertion mutations were deleterious in both the intact and soft rot tomatoes, with ∼300 individual mutations causing growth to a significantly lesser extent (FDR, <0.05) than the wild type (Table S1). Within intact tomatoes, ∼220 mutations were deleterious, while only ∼50 mutations were deleterious in soft rots. Figure 3 is a graphical representation of these findings with pathways downloaded from the Kyoto Encyclopedia of Genes and Genomes (KEGG). While fatty acid biosynthesis appears to be necessary under both conditions, their degradation seems important only in the intact fruit. The tricarboxylic acid cycle (TCA) also plays a role in both conditions, either directly by providing nutrients for Salmonella growth or indirectly through impacting energy yields. However, the conversion of oxoglutarate to succinyl-coenzyme A (succinyl-CoA) and the two-step conversion of fumarate to oxaloacetate were more important in the intact tomato. This supports previous evidence that *S*. Typhimurium 14028 is capable of using alternate metabolic pathways in the soft rot (24). For the most part, with the exception of the mannose-6-phosphate isomerase mutant (*manA*), mutants impaired in glycolytic pathways were less fit in intact fruit. Additionally, with the exception of *rfaF*, which was less competitive in both the soft rot and intact tomatoes, mutants in the lipopolysaccharide (LPS) genes were less competitive in the intact tomato than in soft rot. Finally, we observed that the mutations in nucleotide biosynthesis pathways were the only ones that resulted in the enhanced growth within soft rot. These data show that *S*. Typhimurium 14028 does not use the same essential functions in soft rot and intact tomato tissue.

### Nucleotide synthesis is required for growth of Salmonella in soft rot.

**FIG 4:**
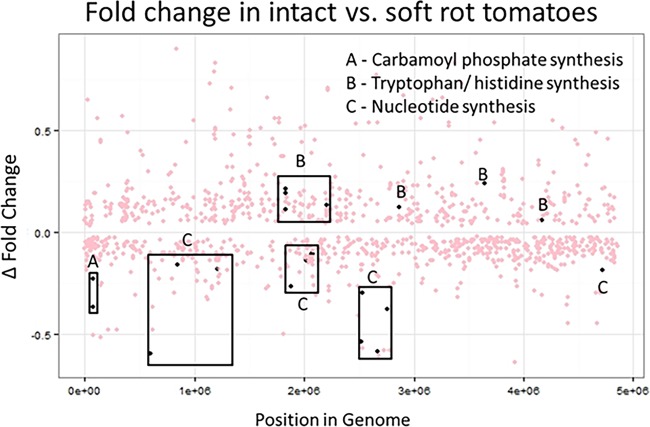
Comparison of genes more or less fit in the soft rot and intact tomatoes. Dots represent significant differences (FDR, <0.05) in the fold change of mutants grown in both tomatoes with soft rot and with no sot rot. Negative values indicate the mutant is at a disadvantage, while positive values indicate a benefit. Black dots are genes which belong to a grouping (A, B, or C) and also cluster on the positive or negative side of the graph.

**FIG 5:**
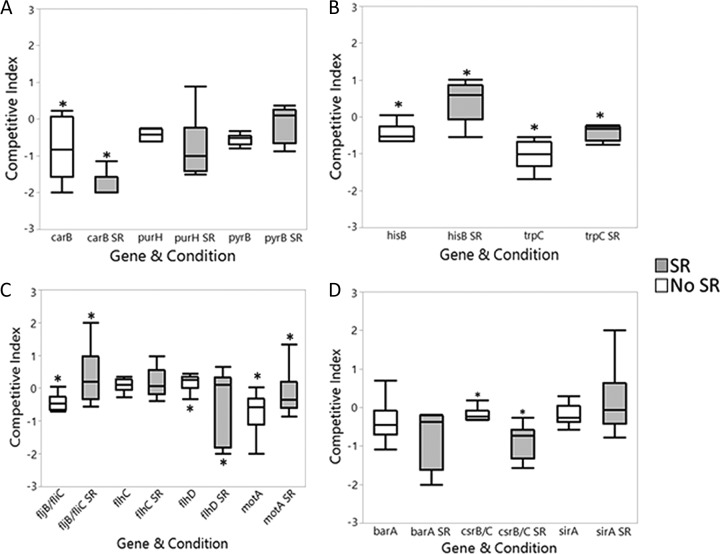
Competition assays in soft rotted and intact tomatoes. Assays were performed by first inoculating the tomatoes with 1:1 “in” ratio of wild type to mutant strains. After 3 days, the bacteria are harvested and grown on XLD agar at 42°C. The colonies were then patched onto LB with antibiotic to establish the “out” ratio of wild type to mutant. The log_10_ of the out ratios of tomatoes with no soft rot (no SR) and those with soft rot (SR) were then compared. Analysis was performed using Student's *t* test. Asterisks signify statistical differences (*P* < 0.05). Genes tested were those involved in nucleotide synthesis (A), amino acid synthesis (B), motility (C), and regulation (D).

Nucleotide synthesis is a necessary step in the replication of DNA and cellular growth. We observed that mutants in genes involved in *de novo* nucleotide synthesis cluster on the negative side of the log scale (Fig. 4). This indicates that mutations in genes involved in purine and pyrimidine synthesis impart a significant disadvantage against the wild-type strain in the soft rot. Among these genes are the *pyr*, *pur*, and *car* genes. The *pyr* and *pur* genes are involved in pyrimidine and purine synthesis, respectively, while the *carAB* gene cluster is involved in the synthesis of arginine and pyrimidines. While the screen results indicate that control of purine and pyrimidine synthesis is important for Salmonella persistence in the soft rot, the individual competition assays showed no significant loss of fitness due to these mutations (Fig. 5A). However, the loss of fitness by *carB*, which is controlled by purine and pyrimidine levels (38), and the results support the notion that nucleotide synthesis is important.

### Amino acid synthesis is not required for growth in the soft rot.

*De novo* amino acid synthesis is important in environments where adequate nutrients are not supplied. It has been demonstrated that in seedlings and ripe tomatoes, Salmonella spp. require *de novo* amino acid synthesis (39, 40). As shown in Fig. 4, among the many mutants that outperformed the wild type in the soft rot were mutants of genes that were necessary for amino acid synthesis, such as histidine and tryptophan, as well as a GDP 3′-diphosphate (ppGpp), which may affect histidine regulation (41). The results of our screen revealed that mutants deficient in the *de novo* synthesis of amino acids, such as those of histidine (*his*) and tryptophan (*trp*), outcompeted the wild type in soft rotted tomatoes (Fig. 4). The results of the individual competition assays with *hisB* and *trpC* mutants supported these findings (Fig. 5B). Carrari et al. have shown that amino acid concentrations are not fully depleted in mature green tomatoes (42). Therefore, the levels of these amino acids are likely high enough in the soft rot that Salmonella spp. are able to scavenge them from the environment and thus avoid the metabolic load of their synthesis.

### Control of motility is important in soft rots but not through BarA-SirA.

Motility is a well-known virulence factor in Salmonella species (43, 44). Taxis toward energy sources has been established as a necessity in the inflamed intestine (45, 46). In cilantro soft rots, it has been demonstrated that genes involved in motility are downregulated (7). Our screen identified a number of genes involved in motility and its regulation as being important for soft rot colonization. To separate the consequences of regulatory and structural mutations, we tested phenotypes of mutants lacking the motor (but with an intact, although nonfunctional, flagellum), flagella (but with a functional motor), as well as a number of regulatory mutants that have decreased motility. The competitive advantage gained by nonmotile mutants suggests that a loss of function of the motor via *motA* and loss of the flagella in the *fljB fliC* mutant (Fig. 5C) provide a benefit in the soft rot. The competitive fitness of mutants in genes known to regulate motility revealed that the deletion of *csrB* and *csrC*, two small regulatory RNAs that regulate the function of the RNA-binding protein CsrA (33), resulted in reduced fitness of Salmonella spp. in the soft rot (Fig. 5D). However, the deletion of the two-component system BarA/SirA, which regulates the expression of the global Csr regulatory system (47–49), had a minimal effect on the fitness of *S*. Typhimurium 14028 in macerated tomato tissue (Fig. 5D). However, the deletion of *flhD*, but not *flhC*, which both function to regulate flagella (among other genes) (50–52), was deleterious (Fig. 5C). This is in line with the fact that the *flhDC* operon is controlled by CsrA (48, 53) and our finding that *csrB* and *csrC* increase *S*. Typhimurium 14028 growth in soft rot. Thus, motility appears to impede soft rot colonization by *S*. Typhimurium 14028, being regulated through an unknown regulatory cascade that does not directly involve CsrA.

## DISCUSSION

Salmonella enterica serovar Typhimurium is highly adaptable and, therefore, capable of survival in a broad range of hosts. The ability of non-typhoidal Salmonella spp. to scavenge nutrients in plant and animal hosts is key to their ability to establish within diverse niches (4, 54–57). Their ability to exploit increased oxygen levels resulting from the depletion of clostridia in animals, to exclusively utilize nutritional resources under anaerobic conditions during cocolonization of macerated leaf tissue with *D. dadantii*, and to inhibit native microbiota by acidifying the environment in plants are common examples of this adaptability (5, 7, 58, 59). While there are instances in which native microbiota inhibit Salmonella proliferation (60, 61), non-typhoidal Salmonella spp. also benefit from the host native microbiota. The presence of bacteria and fungi that degrade plant tissues commonly increased Salmonella cell numbers in/on plants by at least 10-fold in laboratory studies (3, 6, 7, 62). The uptake and catabolism of a broad range of nutrients released from the degraded plant tissue, or made available through its degradation, partly enable this growth enhancement (7, 9). Here, we highlight the impact of *de novo* amino acid synthesis, nucleotide synthesis, and motility on the colonization of tomato soft rot by *S*. Typhimurium 14028.

Competition for amino acids in plant environments may be more critical for bacterial growth than that for carbohydrates, although cross-feeding in microbial communities can occur (63, 64). Contrary to previous reports that *de novo* amino acid synthesis is required for the growth of Salmonella spp. in intact plants (39, 40), soft rot conditions appeared to alleviate this need, as revealed by the competitive advantage gained by auxotrophic mutants in this study. Whether Salmonella spp. can scavenge amino acids and outcompete Pectobacterium spp. or whether other unknown interactions eliminate the need for amino acid synthesis is unclear. Given that Salmonella spp. have the ability to metabolize nutrients that do not serve as substrates in native bacteria in a variety of hosts (3), including in soft lesions of cilantro and lettuce (7), it is possible that they derive amino acids from metabolic pathways particular to their growth in soft rot, rather than through energetically expensive *de novo* synthesis.

Despite differences in growth requirements by Salmonella spp. in plant soft rot and animal hosts, there are also significant similarities. Goudeau et al. (7) demonstrated a considerable overlap in genes upregulated in cilantro and lettuce soft rot, and in the animal intestine, with 76% of the genes involved in metabolic processes. Similarly, purine and pyrimidine synthesis have both been shown to be required for Salmonella colonization of mice, pigs, and red tomatoes (39, 65, 66). Our study revealed that mutations in pyrimidine and purine synthesis along with those in *carB* are also attenuated in the soft rot. Despite individual *pyr* and *pur* mutants not showing a significant disadvantage against the wild type in the competition assays, a negative trend can be seen, as revealed in the data analysis. Mutations in *carB*, which is regulated by pyrimidine and purines (38), showed the greatest attenuation in the soft rot. The performance of this mutant, which is blocked at an intermediate step in pyrimidine biosynthesis, suggests that pyrimidine biosynthesis is in fact necessary in the soft rot (67). Although *carB* plays a role in motility and biofilm formation in Xanthomonas spp., the competitive advantage of the nonmotile mutants that we observed in soft rot suggests that it performs dissimilar functions under the two conditions (68).

It is well established that Salmonella motility is required for virulence in mouse models (45, 46, 69), although it does not appear to be necessary in macrophages (70). Our finding that motility is disadvantageous in tomato soft rot compared to intact tomatoes is surprising but consistent with the previously described transcriptome of Salmonella Typhimurium in cilantro and lettuce leaf macerated by a closely related soft rot pathogen, *D. dadantii* (7). This may be due to increased nutrient availability, which reduces the need for taxis toward substrates, or to the initially abundant presence of cellulose, which can inhibit the movement of flagella (71). Alternatively, the energy conserved by the lack of motility or flagellar synthesis could result in increases in biomass.

The effect of mutations in the pathways regulating flagellar synthesis indicates that there is a concerted effort to respond to the environment generated by soft rot. Despite *flhC* and *flhD* functioning together to activate flagellar genes (51), only the *flhD*-deficient mutant was less competitive against the wild type, while the *flhC*-deficient mutant remained neutral. The ability of FlhC or FlhD to regulate other pathways to include those involved in respiration and cell division may offer an explanation about the inconsistent phenotype imparted by these mutations (71–73). Mutations in *flhD* (but not *flhC*) have been shown to lead to an increased rate of cell division, especially prior to the stationary phase, and to contribute to higher cell numbers (but not biomass) in the stationary phase, which could also explain the phenotypes seen here (74). It is noteworthy that FlhC/D also regulates genes of the Entner-Doudoroff pathway, which are shown to be important in growth of *S*. Typhimurium 14028 in tomato soft rot (24, 72). The loss of fitness by the disruption of *flhD* supports previous evidence that this pathway is involved in the growth of *S*. Typhimurium 14028 in the soft rot (24). In addition, *csrB* and *csrC*, which antagonize CsrA (a positive regulator of the *flhC-flhD* complex), were also attenuated in the soft rot (53, 75). The phenotypes of both the *flhD* and *csrB csrC* mutants are consistent with our observation that motility is disadvantageous to *S*. Typhimurium 14028 in the soft rot. Finally, it is worth noting that while a *csrB csrC* mutant was less competitive in the soft rot, the SirA and BarA mutants had no significant advantages or disadvantages. The BarA/SirA two-component system regulates motility through the *csr* regulatory mechanism (48).

The remarkable adaptability of non-typhoidal Salmonella spp. has likely contributed to their prominence among foodborne pathogens. Whether it is produce, meat, or poultry, there are few foods that are not susceptible to invasion by these pathogens. It is clear that non-typhoidal Salmonella spp. are well equipped to survive environments ranging from the harsh conditions in the phyllosphere to the acidic environments of tomatoes and macrophages. The work presented here sheds light on the intricate relationships that may occur throughout interspecies relationships and how non-typhoidal Salmonella spp. are able to effectively adapt to changing environments.

## MATERIALS AND METHODS

### Strain construction.

Deletion mutants were constructed using Datsenko-Wanner mutagenesis (76). Primers (Table 1) were designed to replace the entire open reading frame (ORF) (from the start to stop codons) with an *frt-kan-frt* cassette (76). Deletions were confirmed by PCR with the primers listed in Table 2. RIVET reporters were constructed by first removing a kanamycin resistance marker and then mating the plasmid pCE70 or pCE71 containing a *tnpR-lacZ* fusion into the mutant strain. The orientation of the insert was confirmed by PCR with primers upstream of the start site and downstream of the stop codon. Phage P22 grown on the Salmonella strain JS246 was then used to transduce a *res-tet-res* marker into the strain with a newly inserted *tnpR-lacZ*. The resulting strains were then purified using EGTA and screened on Evans blue-uranine (EBU) agar, as described previously (24).

**TABLE 1:** Primers used in this study

Primer name	Sequence	Deletion primer
AG131	ATTGAGCAATACCGTCAGTCCGCGAAATAATCAGGAGTAATAAGAGCCTGTAGGCTGGAGCTGCTTCG	*carB* forward
AG132	ACATTATATTACAGGTCCGGTTAGAGCAATATCCGCCGGACCCTTTGTCATATGAATATCCTCCTTAG	*carB* reverse
AG100	CATTCGTGCCAAAAGTGAATAAGTGTGAGCTACTTCAAAGTTGTCAGATGTAGGCTGGAGCTGCTTCG	*gntR* forward
AG101	TCCGCATGTCCGTGGTAAACTGGGCAAATCTATCCCTTTTATACCTTTCATATGAATATCCTCCTTAG	*gntR* reverse
CEC202	TTCTGCAACGCAGGCAGCGTCAGCGTGTGGGTCATTGAGGACGTGTGATGTAGGCTGGAGCTGCTTCG	*amyA* forward
CEC203.1	CTGCCGTAATTTCGCTTCCCGGCAGCGCTCTGCCGCCGGGAACGCTCACATATGAATATCCTCCTTAG	*amyA* reverse
CEC212	CAGTACGGCGACGATACGGTGATGGTCGTCTGGGCGGGCCGCCGCTAATGTAGGCTGGAGCTGCTTCG	*malS* forward
CEC213.1	TTGTTTTGAAGGGGCTACCGGTACGCGAGGAGACCGGTAGCGCCACGACATATGAATATCCTCCTTAG	*malS* reverse

**TABLE 2:** Strains and plasmid used in this study

Strain or plasmid	Genotype or description	Source, construction, or reference
Strains		
Pectobacterium carotovorum subsp. carotovorum		
SR38	Aggressive soft rot pathogen isolated from a shipment of Florida tomatoes	Bender et al. (77)
WPP14	Pectobacterium carotovorum subsp. carotovorum originally isolated from a diseased potato in Wisconsin	Gift from Amy Charkowski at University of Wisconsin-Madison
CCPcc 11	WPP14 *outS*::*frt*-*cam-frt*	This study
Salmonella enterica serovar Typhimurium		
14028	Wild type	American Type Culture Collection
BA3104	14028 *fljB*::*mudJ fliC*::Tn*10*	Iniguez et al. (78)
TIM145	14028 *flhC*::*mudJ*	Teplitski et al. (48)
JS246	14028 *yjeP*::*res1-tetRA-res1*	Datsenko and Wanner (76)
AT351	14028 *flhD*::Tn*10*	Teplitski et al. (48)
AT343	14028 *motA*::Tn*10*	Teplitski et al. (48)
RM6195	14028 *barA*::*kan*	Teplitski et al. (48)
TIM111	14028 *csrB::frt csrC::frt-kan*	Teplitski et al. (48)
BA746	14028 *sirA3*::*cam*	Iniguez et al. (78)
CA774	14028 *ackA-pta::kan*	Lawhon et al. (79)
AG51	14028 *carB*::*frt*-*kan-frt*	Made by Datsenko-Wanner mutagenesis using primers AG131 and AG132
KI3	14028 *gntR*::*frt*-*kan-frt*	Made by Datsenko-Wanner mutagenesis using primers AG100 and AG101
CEC6001	14028 *amyA*::*frt*-*kan-frt*	Made by Datsenko-Wanner mutagenesis using primers CEC202 and CEC203.1
CEC6003	14028 *malS*::*frt*-*kan-frt*	Made by Datsenko-Wanner mutagenesis using primers CEC212 and CEC213.1
CEC5001	JS246 *amyA*::*frt*-*kan-frt*	Made by Datsenko-Wanner mutagenesis using primers CEC201 and CEC203.1
CEC5003	JS246 *malS*::*frt*-*kan-frt*	Made by Datsenko-Wanner mutagenesis using primers CEC211 and CEC213.1
CEC8001	JS246 *amyA*-*frt-kan-frt*	Made by Datsenko-Wanner mutagenesis using primers CEC202 and CEC203.1
CEC8003	JS246 *malS*-*frt-kan-frt*	Made by Datsenko-Wanner mutagenesis using primers CEC212 and CEC213.1
MHM99	14028 *hisB*::*frt*-*kan-frt*	M. de Moraes, unpublished data
MHM73	14028 *trpC*::*frt*-*kan-frt*	de Moraes et al. (39)
MHM89	14028 *purH*::*frt*-*kan-frt*	de Moraes et al. (39)
MHM68	14028 *pyrB*::*frt*-*kan-frt*	de Moraes et al. (39)
Plasmid		
pKD4	FRT-kan-FRT template	Datsenko and Wanner (76)

### Bacterial culture and tomato infections.

Salmonella strains were grown overnight in a shaker at 37°C in Luria broth (LB) supplemented with kanamycin at 50 μg/ml (kan50) for mutants and LB supplemented with tetracycline at 10 μg/ml (tet10) for RIVET reporters. Wild-type Salmonella enterica serovar Typhimurium 14028 was grown without selection. Pectobacterium carotovorum SR38 and WPP14 *outS* mutants were grown overnight in 5 ml of LB broth in a shake incubator at 30°C.

### Tomato infections.

For competitive fitness experiments, overnight cultures of a mutant and the wild-type strains were washed in phosphate-buffered saline (PBS) and diluted to ∼10^4^ CFU/ml. These dilutions were combined in an approximately 1:1 ratio to prepare the inoculum. Three shallow wounds (∼1 mm in diameter, 1 to 3 mm deep) were made in the epidermis of each green tomato. Inocula (3 μl) containing approximately 10 to 100 CFU were injected, for a total of 30 to 300 CFU per tomato. In assays in which Pectobacterium spp. were added to the wounds, an additional 3 μl of an undiluted but washed Pectobacterium suspension was inoculated into the tomatoes, as we have done before (24). Salmonella spp. were harvested after 3 days, as described before (24). Methods and data analyses were carried out as described before (37), with the addition that plates from tomatoes infected with Pectobacterium spp. were incubated at 42°C to reduce the growth of pectobacteria. For total growth experiments, inoculum and infection were performed as described above without the 1:1 ratio.

### Transposon insertion library construction, screening, and sequencing.

Construction of the MZ1597 library of Tn*5* insertion mutants in *S*. Typhimurium 14028 using the Epicentre EZ Tn5<T7/Kan2> promoter insertion kit was described previously (39). The library was screened in green tomatoes inoculated with *S*. Typhimurium 14028 only, the Salmonella library and the wild-type Pectobacterium carotovorum, and the Salmonella library with an *outS* mutant of *P. carotovorum*. Prior to the inoculation, MZ1597 cultures were grown (with shaking at 250 rpm) for 16 h in LB broth supplemented with kanamycin at 37°C. Cultures of *P. carotovorum* were grown under the same conditions at 30°C. The cultures were pelleted, washed in PBS twice, and diluted 1:10, reaching a final density of approximately 10^8^ CFU/ml. Three microliters of the suspension of MZ1597 was inoculated into three shallow (2 to 3 mm deep, 1 mm in diameter) wounds in tomato pericarps (∼10^6^ CFU per tomato); when added, *P. carotovorum* cultures were added in approximately 10-fold excess. Tomatoes were incubated at 22°C for 3 days until signs of the soft rot were fully visible in tomatoes inoculated with the wild-type *P. carotovorum*. Salmonella spp. were recovered by collecting ∼1-g samples of the pericarp around the inoculation site, and samples from the same fruit were combined and homogenized in a stomacher (Sevard). Salmonella cells were recovered by centrifugation and resuspended in 50 ml of LB broth followed by 6 h of growth at 37°C and 250 rpm, reaching ∼10^8^ CFU/ml. One milliliter of culture was recovered and used for library preparation.

### Barcode mapping.

Aliquots of around 5 × 10^7^ CFU from input and output libraries were subjected to three washes in water, followed by proteinase K digestion, as described previously (39). After inactivation of the enzyme, a nested PCR regimen was performed to amplify the DNA regions adjacent to the barcode, as described before (39). The second PCR introduced standard dual 8-base indexes, which were unique to each sample. Samples were pooled and subjected to QIAquick PCR product purification (Qiagen), according to the manufacturer's recommendation. Illumina sequencing proceeded with custom primers Tn5_EZ_Right_Seq_fixed and Tn5_EZ_Index_Seq_new for a single indexed run with a read length of 25 bases. Barcode trimming, removal of duplicate reads using Picard tools, and mapping using Bowtie 2 were carried out as described previously (39). For the identification of barcoded mutants, the raw sequencing data consisted of single-end 25-bp reads. The first 18 bases, which represented the unique N18 tag for each Tn*5* mutant, were extracted, and the abundances of all unique 18-mers were calculated using custom Perl scripts. The abundances of all N18 barcodes mapped within each annotated genome feature were summed in a strand-specific manner. This represented the aggregated abundance for each feature in the coding strand and the noncoding strand. The aggregated abundances for the input and output libraries were statistically analyzed using edgeR, and the log_2_-fold changes and FDRs were reported.

### Metabolic mapping and functional characterization.

Genes required for Salmonella survival in soft rot and intact tomatoes were retrieved from the TIA data set. An FDR of <0.05 and log_2_(fold change) of <0, FDR of <0.05 and log_2_(fold change) >0, and the intersection thereof were used in R studio to assign KEGG Orthology (KO) terms for Salmonella enterica serovar Typhimurium ATCC 14028 coding sequences, and the KEGG Mapper web interface was used to visualize metabolic pathways.

## Supplementary Material

Supplemental material
