# Supplementary material for: Interactions of Salmonella enterica Serovar Typhimurium and Pectobacterium carotovorum within a Tomato Soft Rot
Source: Appl Environ Microbiol. 2018 Feb 14;84(5):e01913-17. doi: 10.1128/AEM.01913-17 (PMC5812938; doi:10.1128/AEM.01913-17)
Supplement: Supplemental material [file supp_84_5_e01913-17__index.html]

Supplemental material 

# Interactions of Salmonella enterica Serovar Typhimurium and Pectobacterium carotovorum within a Tomato Soft Rot

## Supplemental material

- Supplemental file 1 -

  Tomatoes inoculated with *Pectobacterium carotovorum* WPP14 *outS* (Fig. S1); *Salmonella* metabolic pathways in intact tomatoes and tomatoes inoculated with the *outS* mutant (Fig. S2); *Salmonella* genes under selection in tomato fruit, with and without soft rot (Fig. S3); KOs (Table S1).

  PDF, 1001K
