# Supplementary material for: Interactions of Salmonella enterica Serovar Typhimurium and Pectobacterium carotovorum within a Tomato Soft Rot
Source: Appl Environ Microbiol. 2018 Feb 14;84(5):e01913-17. doi: 10.1128/AEM.01913-17 (PMC5812938; doi:10.1128/AEM.01913-17)
Supplement: Supplemental material [file AEM.01913-17_zam005188344s1.pdf]

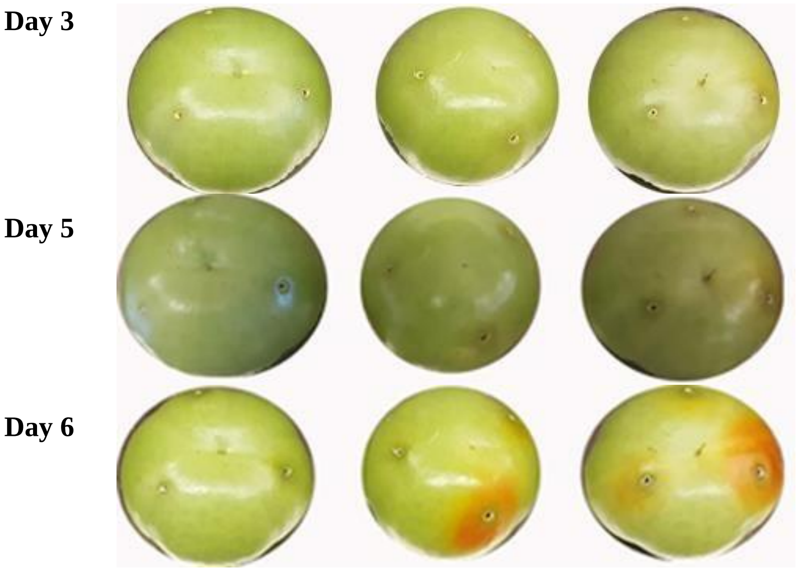

<u>Fig S1. Tomatoes inoculated with *Pectobacterium carotovorum* WPP14 *outS*</u>. Pictures were taken at 3,5, and 6 days post infection. The characteristic water-soaked lesions of a bacterial soft rot are not present at any time point.

Fig S1. Tomatoes inoculated with *Pectobacterium carotovorum* WPP14 *outS*. Pictures were taken at 3,5, and 6 days post infection. The characteristic water-soaked lesions of bacterial soft rot are not present at any time point

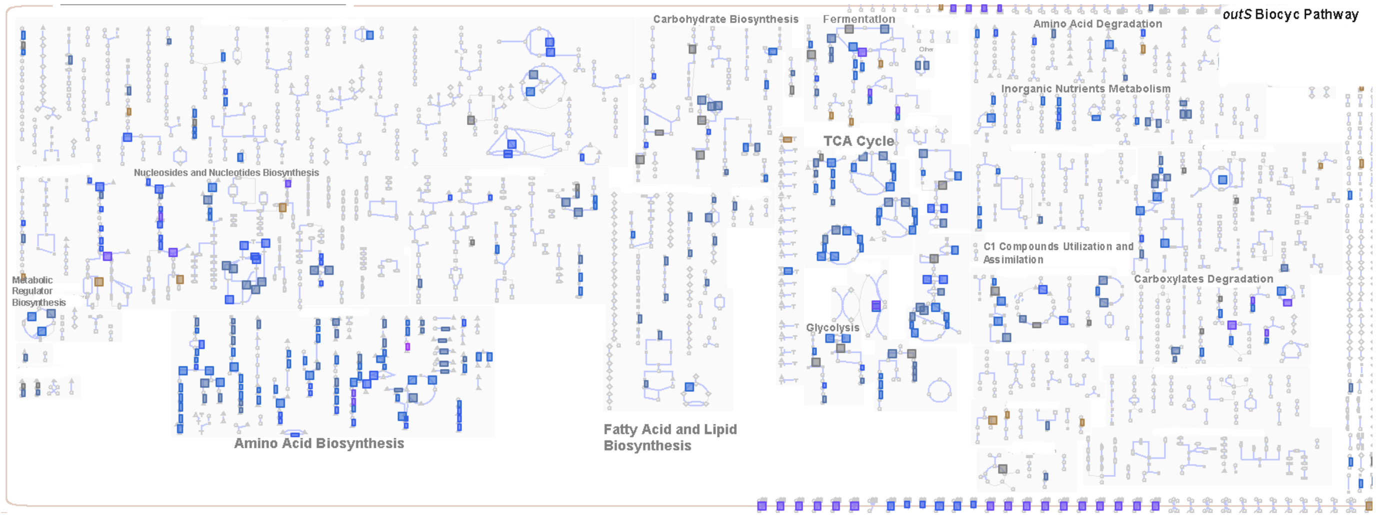

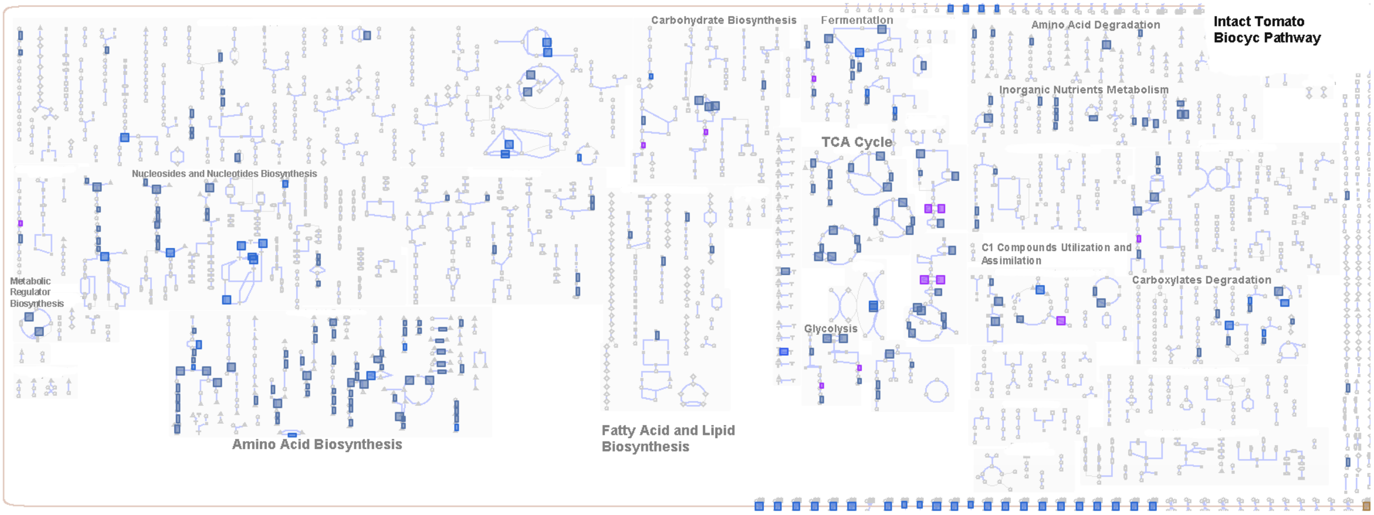

<u>Fig S2. Salmonella metabolic pathways in intact tomatoes and tomatoes inoculated with outS mutant.</u> Map downloaded from Biocyc and color coded to represent mutations that are beneficial (positive values) or harmful (negative values) in the intact tomato and tomato infected with WPP14 outS, a mutant unable to excrete PCWDEs. Boxes which are not colored were either insignificant (FDR <0.15) or not represented.

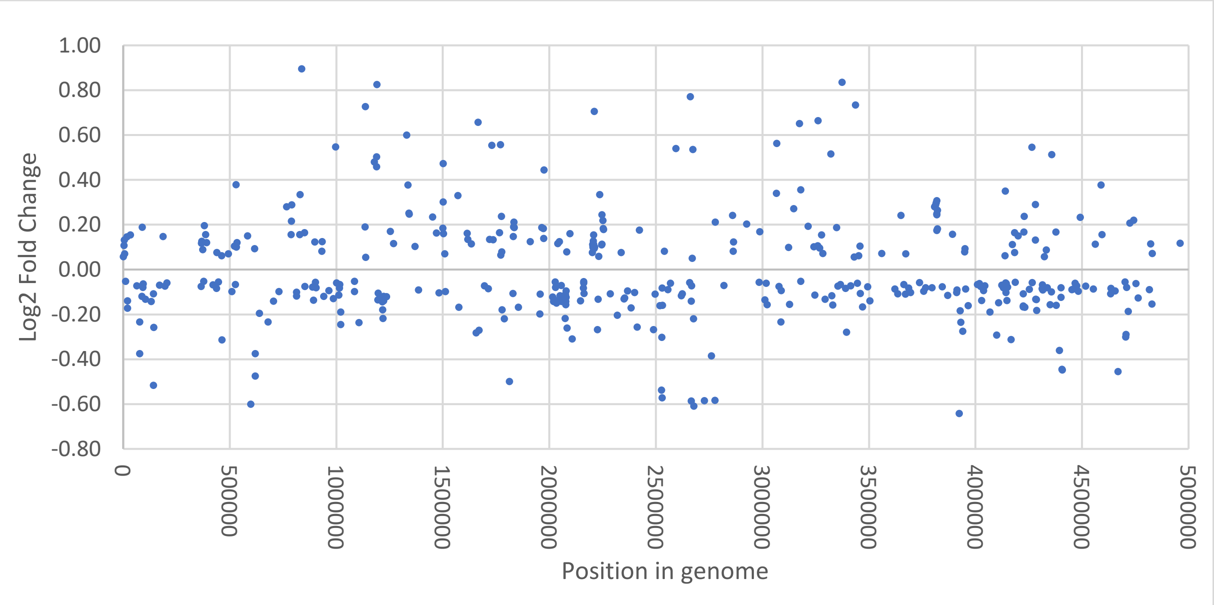

<u>Fig S3. Salmonella genes under selection in tomato fruit, with and without soft rot.</u> Dots represent significant differences in the growth of mutants (FDR <0.5) in order to identify which mutants may be affected by growth in the tomato regardless of the presence of the soft rot. Positive values indicate a mutation that benefits from growth in the tomato while negative values indicate a hindrance.

	Shared KOs	Intact KOs	Pecto Kos
1	K12524	K01924	K03580
2	K00872	K03531	K03925
3	K01733	K03070	K03587
4	K03101	K05799	K05365
5	K01956	K01673	K02072
6	K01955	K01579	K00759
7	K01525	K01265	K07107
8	K03771	K00806	K01468
9	K01704	K00748	K01479
10	K01703	K01962	K11534
11	K00052	K03273	K00656
12	K01649	K08224	K00813
13	K05806	K07345	K03621
14	K03438	K03417	K03327
15	K03586	K03210	K01809
16	K13301	K03151	K01420
17	K01682	K04752	K15580
18	K00097	K03320	K04072
19	K00970	K01816	K03609
20	K00990	K02083	K03607
21	K07277	K03269	K00978
22	K06142	K01491	K00339
23	K02073	K07214	K06076
24	K02071	K02361	K11175
25	K01270	K01252	K09159
26	K00931	K03386	K00605
27	K00147	K03387	K05596
28	K00286	K01646	K02600
29	K14591	K01910	K03624
30	K07568	K06217	K07301
31	K00773	K03184	K03570
32	K03072	K09690	K03076
33	K03074	K09691	K04759
34	K07738	K00240	K00705
35	K05839	K00164	K07473
36	K18138	K00426	K01534
37	K03585	K03562	K02841
38	K06187	K01678	K02840
39	K01589	K01677	K02844
40	K01588	K07404	K00762
41	K03768	K03521	K03718
42	K02364	K03522	K05790
43	K02363	K00249	K02472
44	K00216	K00311	K08318
45	K06189	K05844	K02548
46	K07042	K01620	K03591

47	K01953	K03694	K00928
48	K02565	K03632	K04068
49	K01443	K03310	K00527
50	K00241	K09857	K01779
51	K00242	K04770	
52	K00239	K13085	
53	K01903	K18997	
54	K01902	K01788	
55	K00425	K04338	
56	K01834	K04337	
57	K01784	K04333	
58	K16013	K04335	
59	K03719	K02517	
60	K03466	K08300	
61	K00831	K02911	
62	K00800	K00648	
63	K00763	K00645	
64	K00254	K02778	
65	K01465	K02779	
66	K06179	K03723	
67	K07040	K09808	
68	K09458	K15347	
69	K07660	K11071	
70	K07153	K11072	
71	K01222	K03704	
72	K06078	K02032	
73	K00873	K11250	
74	K03683	K02760	
75	K03612	K02761	
76	K09475	K02759	
77	K19226	K03490	
78	K19227	K13695	
79	K13634	K19709	
80	K06178	K18457	
81	K01657	K14978	
82	K13497	K03220	
83	K13498	K15345	
84	K01696	K03222	
85	K01695	K03230	
86	K06190	K03226	
87	K15581	K03229	
88	K07001	K07390	
89	K02493	K06938	
90	K06942	K03614	
91	K01297	K03615	
92	K03603	K03616	
93	K03611	K03617	

94	K03610	K01488
95	K03608	K14062
96	K03684	K11744
97	K03797	K11743
98	K01625	K19778
99	K00036	K00027
100	K03551	K02029
101	K03550	K02028
102	K01159	K12262
103	K08310	K14055
104	K02407	K16052
105	K02422	K19228
106	K02423	K19230
107	K00765	K03113
108	K00013	K10823
109	K00817	K02485
110	K01089	K09858
111	K02501	K07300
112	K01814	K00948
113	K00523	K01194
114	K01079	K02795
115	K11733	K07313
116	K00882	K01354
117	K03734	K19337
118	K00925	K19304
119	K13788	K02500
120	K00764	K11755
121	K03558	K00012
122	K06173	K00033
123	K01736	K16711
124	K07320	K03818
125	K08296	K13620
126	K01738	K16692
127	K02045	K01104
128	K02047	K01991
129	K02046	K07289
130	K02048	K07347
131	K01923	K03593
132	K01933	K06518
133	K00937	K03748
134	K03601	K17723
135	K06941	K10542
136	K13628	K01495
137	K04488	K07014
138	K00600	K07687
139	K03596	K00343
140	K03803	K03475

141	K03088	K07052
142	K05590	K00647
143	K06180	K08566
144	K14187	K11535
145	K01626	K01885
146	K01919	K03528
147	K03553	K04031
148	K00860	K06957
149	K00956	K03548
150	K00957	K10763
151	K00390	K01951
152	K00381	K04755
153	K00380	K04044
154	K00951	K04487
155	K14682	K13643
156	K03582	K04751
157	K08311	K08177
158	K01586	K03584
159	K07462	K03595
160	K04763	K03100
161	K18800	K14170
162	K01262	K03106
163	K00058	K07173
164	K00615	K02468
165	K01480	K15831
166	K01920	K02078
167	K02428	K03228
168	K01760	K04058
169	K07645	K03219
170	K12340	K03583
171	K02858	K13292
172	K03086	K02437
173	K01754	K01585
174	K05592	K06997
175	K05803	K03561
176	K01940	K03272
177	K03270	K00982
178	K05808	K09979
179	K02806	K03979
180	K06958	K09774
181	K07648	K06861
182	K00265	K00024
183	K00266	K03500
184	K09908	K03499
185	K03402	K02931
186	K08301	K02965
187	K18765	K02355

188	K05540	K02992
189	K03557	K10914
190	K00014	K00891
191	K13638	K07638
192	K02358	K01179
193	K03775	K00009
194	K00821	K00057
195	K06141	K03071
196	K02302	K02843
197	K01867	K00556
198	K01783	K01624
199	K06223	K02774
200	K03112	K02773
201	K01735	K02111
202	K07400	K05851
203	K02444	K03183
204	K00111	K03116
205	K00703	K03182
206	K06145	K04564
207	K16322	K01776
208	K15984	K03524
209	K00383	K02601
210	K02562	K02863
211	K00640	K09891
212	K02848	K00631
213	K00989	K00832
214	K01139	K12542
215	K03655	K12541
216	K07507	K04084
217	K08154	K04077
218	K03629	K19810
219	K03650	K03477
220	K02036	K02939
221	K02038	K11737
222	K02037	K03335
223	K02040	K00851
224	K00820	K01154
225	K04042	
226	K03495	
227	K01652	
228	K00826	
229	K01687	
230	K02521	
231	K00053	
232	K01524	
233	K03732	
234	K03671	

235	K01791
236	K16704
237	K02805
238	K16693
239	K12582
240	K02852
241	K02496
242	K01778
243	K09921
244	K03733
245	K03657
246	K03284
247	K03576
248	K00549
249	K03690
250	K03117
251	K03118
252	K05368
253	K03498
254	K03673
255	K02335
256	K07712
257	K07708
258	K01915
259	K06207
260	K07640
261	K13283
262	K00850
263	K03710
264	K11531
265	K08321
266	K01739
267	K12525
268	K00297
269	K01595
270	K01438
271	K00145
272	K00930
273	K01755
274	K04761
275	K00322
276	K01945
277	K00602
278	K00651
279	K00901
280	K00246
281	K00245

282	K04568
283	K04088
284	K04087
285	K01939
286	K13771
287	K12573
288	K03218
289	K01082
290	K09889
291	K09022
292	K00609
293	K00611
294	K09893
295	K01255
296	K13637
297	K02344
298	K02837
299	K03720
